# Running the Numbers: High Metabolic Load Distance as an Alternative to Traditional External Load Metrics in Elite Women's Football

**DOI:** 10.1002/ejsc.70288

**Published:** 2026-11-13

**Authors:** Joshua D. Waugh, Kate L. Pumpa, Celeste E. Coltman, Nick Ball, Bradley Clark, Jocelyn K. Mara

**Affiliations:** ^1^ Research Institute of Sport and Exercise University of Canberra (UCRISE) Canberra Australia; ^2^ Institute for Sport and Health University College Dublin Belfield Ireland; ^3^ Grounded Data Science Canberra Australia

**Keywords:** athlete monitoring, athlete wellness, training load

## Abstract

This study examined the relationship between High Metabolic Load Distance (HMLD) and both session RPE (sRPE) and athlete wellness in women's football and compared it with traditional load monitoring metrics. An observational design was used to collect data from one elite women's football team during training and games in the 2024/25 A‐League W season. Twenty‐four athletes wore global positioning devices to capture external load metrics, including total distance (TD), high‐speed running distance (HSRD) and HMLD. Session RPE and daily wellness were collected through online questionnaires. Generalised additive models were used to examine non‐linear relationships between external load, subsequent days' wellness and RPE, and relationships between morning wellness and same‐day external load. Higher load was associated with increased sRPE, and poorer next‐day wellness, with effects diminishing within two to 3 days post‐session. Across all outcomes, HMLD demonstrated stronger and more consistent relationships than TD and HSRD, explaining a greater proportion of variance in wellness and sRPE. Same‐day wellness was associated with differences in distances, with higher motivation and lower fatigue corresponding to higher HMLD. HMLD demonstrated comparatively stronger associations with immediate perceived exertion and short‐term athlete reported wellness than traditional distance‐based metrics in this cohort. These findings support the application of HMLD as a practical and sensitive measure to inform training decision‐making in elite women's football.

## Highlights

- High metabolic load distance (HMLD) showed stronger associations with athlete wellness and session RPE than traditional distance‐based metrics.
- The influence of external load on athlete wellness was greatest within the first 2 days following training and matches, diminishing by day 3.
- Higher motivation and lower fatigue were associated with greater HMLD on the same day.
- Findings support the use of HMLD as a complementary metric for athlete monitoring in elite women's football.

## 1 Introduction

Training load management is an essential component of performance optimisation and injury prevention in elite women's football (Winther et al. 2024). Practitioners commonly use wearable global positioning system (GPS) devices to monitor external loads, focusing on metrics such as total distance (TD) and high‐speed running distance (HSRD) as standard indicators of training load (Rago et al. 2020). However, there is growing interest in more comprehensive approaches, including high metabolic load distance (HMLD), a metric that integrates high‐speed running distance with accelerometer‐derived energetic cost. Depending on the GPS/GNSS manufacturer and model, modern athlete tracking devices can generate in excess of 40 external load variables per session (Heyworth 2023). These variables typically include locomotor measures derived from positional data (e.g., TD, speed‐zone distances and velocity‐based measures) and mechanical metrics derived from embedded inertial sensors (e.g., accelerations, decelerations, impacts and composite mechanical load measures) (Bourdon et al. 2017). Despite this breadth, practitioners typically rely on a core group of measures– TD, HSRD, sprint distance, session maximum velocity, percentage of season maximum velocity, acceleration, deceleration and sprint counts, and relative distance– to interpret training and match demands (Bourdon et al. 2017). However, managing and effectively integrating multiple metrics can be burdensome and increases the likelihood that significant changes may be overlooked or misinterpreted (Dalen et al. 2016). A more reductive approach, which consolidates key aspects of load into fewer and more comprehensive indicators, may therefore be advantageous for decision‐making processes.

Traditional distance‐based measures that emphasise volume and speed are practical and widely used; however, they often fail to capture high‐intensity actions such as accelerations, decelerations and change of direction efforts. These explosive movements impose substantial neuromuscular strain, and contribute to fatigue accumulation (Harper et al. 2019; Harper and Kiely 2018; Tan et al. 2025b), yet may be underrepresented by traditional load metrics (Rhodes et al. 2021), meaning a significant proportion of mechanical and energetic demand may be missed. This discrepancy is meaningful given the increasing emphasis on understanding the dose‐response relationship in football, defined here as the relationship between the magnitude of external load exposure and subsequent perceptual, physiological, fatigue, readiness and recovery responses (Ellis et al. 2021). Metrics that better reflect energetic and mechanical stresses may therefore improve our understanding of how external load translates to internal response.

High metabolic load distance offers a physiologically meaningful representation of high‐intensity movement demands, by integrating speed changing actions into a metabolic cost (Abbott et al. 2018). However, as HMLD is derived from overlapping locomotor behaviour, captured by traditional external metrics, comparisons between these measures should be interpreted as comparative representatives of external demand, rather than independent physiological constructs. In Catapult Sports GPS systems, HMLD represents the distance covered when instantaneous metabolic power exceeds 25.5 W/kg (Wilson 2024), accounting for individual body mass, and improving the ecological validity relative to distance‐based measures (Smith et al. 2019). This approach is based on the metabolic power model proposed by Di Prampero et al. (2005), which estimates the energetic cost of accelerated running on flat terrain, by modelling it as energetically equivalent to running uphill at a constant speed. This framework was adapted for intermittent team sports, forming the basis of metabolic power‐derived external load metrics such as HMLD (Osgnach et al. 2010). While HMLD provides an applied summary of high‐intensity running demands by integrating speed‐ and acceleration‐derived energetic cost estimates, the validation of metabolic power thresholds in women's football remains limited and requires further investigation.

Research in men's football suggests that HMLD differentiates positional demands more sensitively than TD, with greater discrepancies and higher HMLD values observed between central and wide midfielders compared to central defenders (García‐Calvo et al. 2022). Beyond positional profiling, research in men's football and rugby union, comparing metabolic load distance to traditional speed‐ or distance‐based metrics has shown that HMLD captures high‐intensity work that would otherwise be missed by TD or HSRD alone, due to its integration of accelerations and decelerations (Dubois et al. 2017; Gaudino et al. 2013). Furthermore, in men's football, monitoring models that incorporate metabolic‐power distance and acceleration‐based indicators predicted players' session ratings of perceived exertion (sRPE) across a competitive season more effectively than those solely relying on traditional metrics (Vardakis et al. 2024). This suggests that external load derived from metabolic cost may more closely reflect the perceived effects of training experienced by athletes.

External load is typically interpreted alongside internal indicators such as sRPE and daily wellness to assess athletes' perceptual and physiological responses to training (Saw et al. 2016). While sRPE reflects an immediate internal response to the session itself (Foster et al. 2001), wellness captures wider aspects of athlete status, including fatigue, soreness, mood and general wellbeing, which may be influenced by both training and non‐training factors (Gallo et al. 2016). These measures offer insights into fatigue, recovery status and readiness for subsequent load. Comparing the strength and shape of these dose‐response relationships of external load and wellness, and external load and sRPE relationships across different metrics therefore provides an opportunity to determine whether HMLD reflects internal response more effectively than traditional measures.

This study aimed to investigate the use of HMLD in elite women's football and to compare its associations with athlete wellness and sRPE to those observed with traditional load monitoring metrics. The findings from this study may contribute valuable insight to the ongoing development of more effective training load monitoring practices in elite women's football.

## 2 Methods

### 2.1 Experimental Approach to the Problem

This study used an observational single cohort design to investigate HMLD in elite women's football during the 2024/25 A‐League W (Australia's highest level of domestic competition for women's football) season to reflect the full monitored team's environment. This design allowed for the collection of data in regular training and match environments, accounting for natural variability and context‐specific demands, whilst ensuring high ecological validity to high‐performance contexts.

External training load metrics (HMLD, TD, HSRD) were collected using Catapult Vector Core X7 GPS units (Finster‐Rowan 2024). Subjective measures including session RPE and daily wellness were recorded via online questionnaires using the Lumin Sports Athlete Monitoring System (Lumin Sports 2025). Additionally, bodyweight data were measured weekly, 2 days post‐match. Bodyweight data was used to calculate individualised HMLD thresholds each week.

The relationships between each training load metric and wellness on the next day (*T* + 1), wellness 2 days post (*T* + 2), wellness 3 days post (*T* + 3), RPE, and wellness explaining the same day training load data were explored.

### 2.2 Sampling and Participants

Twenty‐four female footballers (*n* = 4 goalkeepers, *n* = 7 defenders, *n* = 6 midfielders, *n* = 7 attackers) were voluntarily recruited from an A‐League W team over the 2024/25 A‐League W season, by use of convenience sampling. Convenience sampling was deemed appropriate due to the accessibility of participants to the researchers and the exploratory nature of the study (i.e. the football team was based in the same city as the research team). Participants had a mean age of 21.8 ± 4.1 years, and mean body mass at the start of the season of 66.4 ± 6.6 kg. Prior to data collection, all participants were briefed verbally and in writing about the nature of the research and provided their informed consent to participate in the study. Participants under the age of 18 prior to the study's commencement also required consent from a parent or guardian to participate in the study. The study was approved by the University's Human Research Ethics Committee (HREC: 14,242).

### 2.3 Data Collection

#### 2.3.1 Anthropometric Testing

Participants' body mass was measured weekly as part of a standardised testing procedure 2 days after a match, using VALD ForceDecks (VALD Performance 2025), prior to weekly countermovement jump testing, with only body mass data collected as part of this study. Body mass was obtained with participants' shoes on, and wearing consistent clothing from week to week, to ensure fluctuations in weight were not associated with different attire being worn. Body mass data were used to calculate an individual's metabolic power threshold each week, and was measured using the VALD ForceDecks for two reasons: (1) to minimise any potential mental health concerns for athletes that may be associated with weekly isolated bodyweight testing (Carrigan et al. 2015), (2) ForceDecks calculate body mass from calibrated vertical ground‐reaction force with good to excellent validity and reliability compared to laboratory‐grade force platforms, with minimal systematic bias (Collings et al. 2024).

#### 2.3.2 External Training Load Data

Participants wore Catapult Sports Vector X7 wearable athlete tracking devices at every training session (*n* = 111) and competition match (*n* = 24) to collect external training load data. These devices were worn between the scapulae in the upper back using a manufacturer‐designed vest underneath the playing jerseys.

Catapult Sports proprietary software, OpenField (Souza 2025), was used to export the running load metrics and consisted of TD (*m*), relative HSRD (*m*, distance covered at > 70% of individual's maximum speed), and HMLD (*m*). All of these distances are calculated internally by the GPS units based on the platform's pre‐defined processing algorithms with individualised thresholds where applicable. HMLD was calculated as the distance accumulated above the manufacturer‐defined threshold of instantaneous metabolic power (25.5 W/kg). User‐level adjustment of this threshold was not available within the platform environment available to the investigative team.

#### 2.3.3 Daily Wellness

Each morning of the competitive season, between 6 AM and 10 AM, or prior to any scheduled team activities in the morning, participants completed a self‐reported wellness questionnaire using the Lumin Sports Athlete Monitoring System on their own smart devices. The questionnaire was adapted from the work of Kellmann and Kölling (2019), and assessed participants' muscle soreness, fatigue, sleep quality, stress, motivation and mood. Responses were provided using a continuous digital slider on a 0‐10 scale, with 0.1 unit increments (Table 1). Descriptive anchor labels were displayed dynamically when participants interacted with the slider to guide interpretation of the response scale. An aggregate wellness score was also calculated as the average of each wellness metric for each participant on each day.

**TABLE 1:** Wellness questionnaire presented to participants.

Wellness metric	Band 1	Band 2	Band 3	Band 4	Band 5
Motivation to train	0–1.9 not at all	2–3.9 less	4–5.9 moderate	6–7.9 more	8–10 very
Muscle soreness^a^	0–2.4 severe	2.5–4.9 moderate	5–7.4 mild	7.5–10 none at all	—
Mood	0–3.3 low	3.4–6.6 normal	6.7–10 fantastic	—	—
Stress^a^	0–2.4 highly stressed	2.5–4.9 moderate	5–7.4 less stressed	7.5–10 No stress	—
Fatigue^a^	0–1.9 total fatigue and exhaustion	2–3.9 very fatigued	4–5.9 moderately fatigued	6–7.9 a little fatigued	8–10 not at all
Sleep quality	0–1.9 severely disturbed	2–3.9 disturbed	4–5.9 average	5–7.9 good	8–10 excellent

*Note:* (a) Questionnaire developed by Lumin Sports, based on research by Kellmann and Kölling (2019).

^a^ Metrics were inverted to ensure results were on the same scale as the rest of the wellness metrics (0 being worst rated, 10 being best).

#### 2.3.4 Session Rating of Perceived Exertion (sRPE)

Within 30 minutes of training or competition matches finishing, participants reported their sRPE for the session using Borg's CR10 scale (Borg 1982), a widely validated measure of internal load in team sport settings (Haddad et al. 2017). Responses were collected individually via an online questionnaire on the Lumin Sports Athlete Monitoring System on participant's personal smart devices, reducing visibility of teammates' responses and minimising social influence (Minett et al. 2021). Standardised instructions were given at the beginning of data collection to ensure participants rated overall session exertion rather than isolated moments of effort.

### 2.4 Statistical Analysis

All analyses were conducted using R version 4.5.0 (R Core Team 2025) in RStudio version 2024.12.1.563 (Posit Team PBC 2025). Initial descriptive and exploratory analyses were performed to examine the variable distribution, identify outliers or missing data, and confirm the suitability of the external load and wellness variables for subsequent analysis.

Internal consistency of the wellness questionnaire was assessed using Cronbach's *α* (*α* = 0.77), indicating that the items in the questionnaire were a coherent construct and were suitable for aggregation into a composite wellness score. To characterise overlap between external load metrics, Pairwise Pearson correlations (*r*) were calculated and visualised in a correlation matrix (Supporting Information S2: Figure S1), alongside variance inflation factor (VIF) assessment. Correlation magnitudes were interpreted using the magnitude‐based thresholds proposed by Hopkins (2002), and VIF values referenced by James et al. (2013). In the present dataset, correlations between external load metrics fell within the large (0.5–0.7) and very large (0.7–0.9) bands, and VIF values were all below the acceptable cutoff of multicollinearity (VIF > 5).

Generalised additive models (GAMs) were used to examine the dose‐response relationships between each external load metric and wellness and RPE outcomes as these relationships are unlikely to follow strictly linear patterns and demonstrate threshold effects and non‐uniform recovery trajectories (Impellizzeri et al. 2019). GAMs provide a flexible modelling framework for estimating such non‐linear relationships without imposing a pre‐defined polynomial structure (Wood 2017). These models were fitted using the mgcv package in R, and model parameters were estimated using restricted maximum likelihood (REML). Separate GAMs were fitted for each metric, with smooth terms applied to capture potential non‐linear effects, and participant ID was included as a random effect to account for repeated observations. Relationships with wellness score the next day (*T* + 1), 2 days after (*T* + 2) and 3 days after (*T* + 3) training were modelled to capture recovery response. For wellness outcomes, a decayed autoregressive term was incorporated for temporal dependency in repeated wellness observations across consecutive days, recognising that subsequent wellness scores are influenced by prior wellness state in addition to recent external load exposure. Session RPE only was modelled using the same‐day load due to its immediate linkage to session demands. In addition, models were created to explore the effect of morning wellness scores on the same‐day external load, reflecting how athlete status may influence training output. To assess whether the distinct locomotor demands of goalkeepers materially influenced model behaviour, all primary analyses were repeated in a sensitivity analysis excluding goalkeepers. The results were similar in both analyses (Supporting Information S2: Figures S2–S5), so goalkeepers were included in the final analysis. An annotated example of the R code used for GAM modelling is provided as an additional file for review (Supporting Information S1: File S1).

Model performance and effect characteristics were summarised using adjusted *R* ^2^, deviance explained, effective degrees of freedom (EDF), F‐statistics, and associated *p*‐values (Table 2). Adjusted *R* ^2^ and deviance explained were used to quantify the proportion of variance captured by each model, accounting for its complexity. Based on guidelines from Moore et al. (2013), adjusted *R* ^2^ values were interpreted using the following thresholds: < 0.3 = negligible, 0.3–0.5 = weak, 0.5–0.7 = moderate, and > 0.7 = strong model fit. EDF values described the degree of non‐linearity in each smooth term, with values greater than one indicating a non‐linear effect. F‐statistic and *p*‐values were used to quantify the strength and statistical significance of each smooth term.

**TABLE 2:** Generalised additive model fit statistics for all load metrics.

Model	Adjusted R‐squared	Deviance explained	Sample size (*n*)	EDF	F‐statistic	*p*‐value
Session + 1 day wellness						
Total distance	0.658	66.2%	3646	7.83	55.96	0.000
High‐speed running distance	0.627	63.1%	3646	2.63	42.49	0.000
High metabolic load distance	0.648	65.2%	3646	3.90	76.30	0.000
Session + 2 days wellness						
Total distance	0.619	62.3%	3622	6.34	15.97	0.000
High‐speed running distance	0.607	61.1%	3622	2.98	1.96	0.110
High metabolic load distance	0.618	62.2%	3622	6.73	14.10	0.000
Session + 3 days wellness						
Total distance	0.617	62.1%	3598	5.13	10.78	0.000
High‐speed running distance	0.618	62.1%	3598	4.05	14.33	0.000
High metabolic load distance	0.617	62.1%	3598	4.05	12.75	0.000
Same day RPE						
Total distance	0.477	49.2%	1089	7.94	73.43	0.000
High‐speed running distance	0.374	39.0%	1089	2.64	106.12	0.000
High metabolic load distance	0.497	51.2%	1089	7.49	82.61	0.000
Wellness to same‐day HMLD load						
Motivation to train	0.215	22.1%	3742	3.20	171.00	0.000
Muscle soreness	0.100	10.6%	3742	3.03	23.48	0.000
Mood	0.094	9.9%	3742	1.00	59.53	0.000
Stress	0.086	9.2%	3742	2.62	7.06	0.000
Fatigue	0.110	11.6%	3742	2.83	35.64	0.000
Sleep quality	0.091	9.7%	3742	3.21	11.48	0.000

Estimated smooth functions, confidence intervals (CI), and effect sizes were extracted to compare the direction and magnitude of each metrics (Supporting Information S3: Table S1). These were reported in the results section as the slope of the function (*β* ± 95% CI). Standard GAM diagnostics such as residual checks and k‐index evaluation confirmed the adequacy of all fitted models.

## 3 Results

### 3.1 Descriptive Statistics

Descriptive statistics for all GPS‐derived external load metrics and self‐reported measures are presented in Table 3. All external load metrics demonstrated substantial variability across sessions, with mean values differing by playing position and session type. sRPE and wellness scores demonstrated comparatively smaller dispersion, reflecting greater consistency in athlete‐reported responses across the season.

**TABLE 3:** Descriptive statistics (mean ± SD) of all external load and self‐reported measures used in the gam analysis.

Position	High metabolic load distance (*m*)	High‐speed running distance (*m*)	Total distance (*m*)	RPE (/10)	Wellness (/10)
Overall					
Central attacker	540 ± 317	134 ± 137	4654 ± 2012	5.4 ± 1.9	7.0 ± 1.3
Central defender	543 ± 314	135 ± 138	4584 ± 2169	5.0 ± 1.8	5.5 ± 0.8
Central midfielder	467 ± 266	128 ± 140	4582 ± 2247	5.2 ± 1.5	6.0 ± 0.8
Wide attacker	542 ± 319	107 ± 109	4348 ± 1853	4.6 ± 1.5	7.3 ± 0.9
Wide defender	586 ± 364	143 ± 143	4787 ± 2523	3.7 ± 1.9	6.0 ± 1.0
Goal keeper	193 ± 145	52 ± 79	2853 ± 1218	4.9 ± 1.7	6.1 ± 1.1
Training					
Central attacker	484 ± 271	116 ± 126	4299 ± 1597	5.4 ± 1.9	6.9 ± 1.3
Central defender	479 ± 265	124 ± 139	4039 ± 1424	5.0 ± 1.8	5.5 ± 0.8
Central midfielder	417 ± 224	116 ± 138	4078 ± 1485	5.2 ± 1.6	5.9 ± 0.8
Wide attacker	497 ± 288	97 ± 108	4064 ± 1432	4.6 ± 1.5	7.2 ± 0.9
Wide defender	476 ± 277	111 ± 129	3902 ± 1498	3.7 ± 1.9	5.9 ± 1.0
Goal keeper	191 ± 133	56 ± 77	2781 ± 1006	4.9 ± 1.7	6.1 ± 1.1
Games					
Central attacker	766 ± 385	206 ± 155	6097 ± 2761	8.0 ± NA	7.5 ± 0.7
Central defender	868 ± 344	192 ± 123	7345 ± 3051	7.0 ± 1.0	5.6 ± 0.9
Central midfielder	722 ± 314	186 ± 136	7146 ± 3430	6.4 ± 0.5	6.5 ± 0.8
Wide attacker	792 ± 365	162 ± 99	5900 ± 2891	6.0 ± NA	7.7 ± 0.6
Wide defender	1029 ± 335	270 ± 125	8364 ± 2668	—	6.4 ± 0.8
Goal keeper	210 ± 210	26 ± 89	3336 ± 2108	5.6 ± 2.2	6.4 ± 1.1

*Note:* Game RPEs for some positional groups were unavailable, due to a lack of observations.

Pairwise correlations between external load metrics ranged from moderate to very large (*r* = 0.57–0.92) and are visualised in Supporting Information S2: Figure 1. HMLD showed very large correlations with HSRD (*r* = 0.76) and TD (*r* = 0.86), indicating overlap between the locomotor constructs captured by the selected external load metrics. However, the VIF values remained below the commonly accepted thresholds for multicollinearity (VIF = 1.42–3.77).

#### 3.1.1 *T* + 1 Wellness Score

On the day after a training session or game, all external load metrics demonstrated negative associations with next‐day wellness, with wellness score decreasing as training load increased (Figure 1). The relationship was strongest for HMLD, which displayed the steepest decline across the load range (*β* = −0.0011, 95% CI [−0.0012, −0.0011]). HSRD also had a pronounced negative effect (*β* = −0.0006, 95% CI [−0.0007, −0.0006]), although smaller in magnitude than HMLD. TD had a much smaller influence on next day wellness (*β* = −0.0001, 95% CI [−0.0001, −0.0001]). Although the above per‐metre estimates seem small, they practically translate to meaningful load changes; for example, a 300m increase in HMLD was associated with a 0.3–0.4 unit reduction in wellness score. Across each of the three metrics, Figure 1B shows clear non‐linear behaviour, with the most pronounced reductions in wellness observed at moderate to high training loads, relative to each metric, with HMLD showing the most pronounced drop compared to the other two metrics.

**FIGURE 1:**
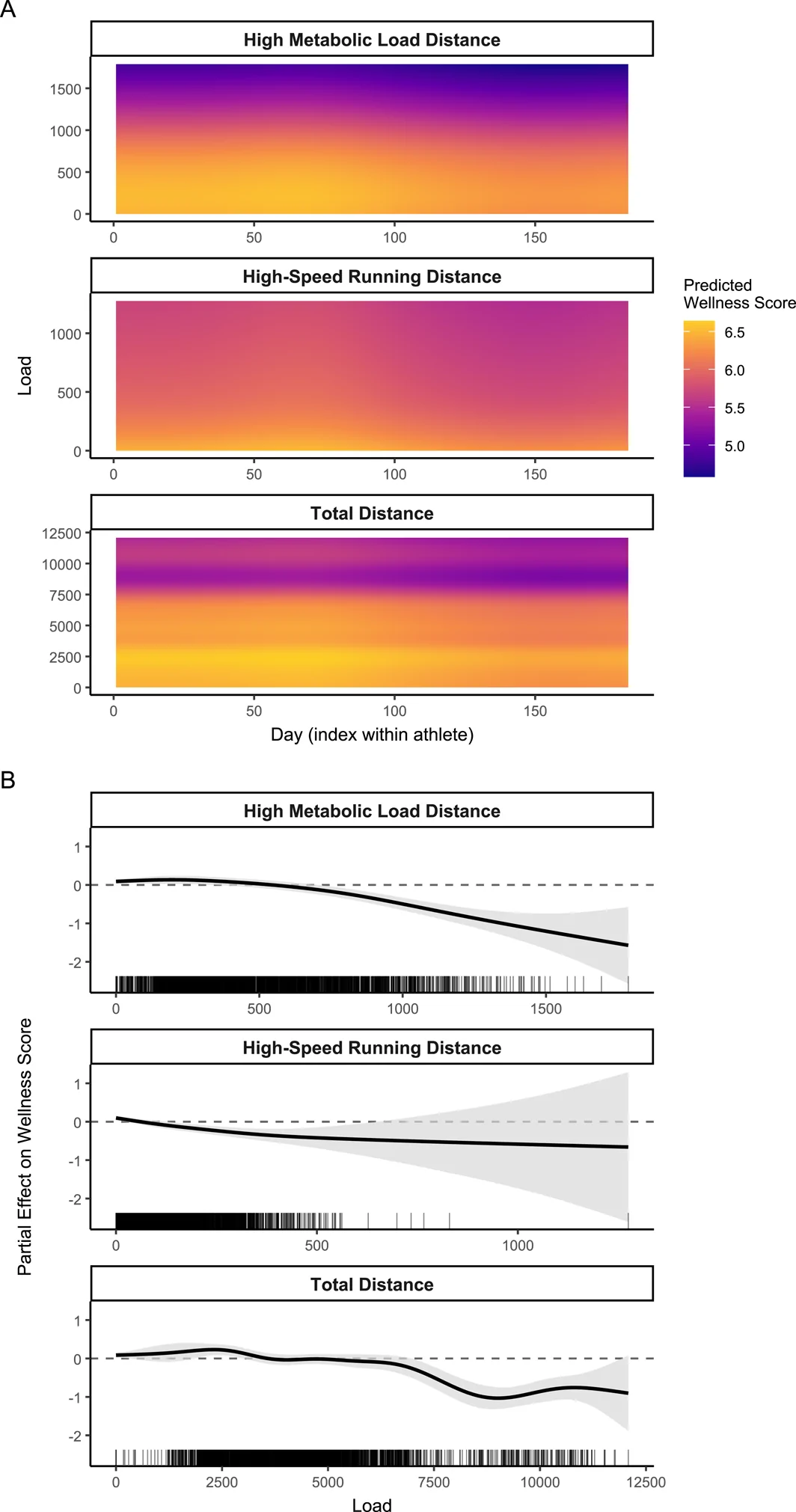
Generalised additive model outputs for *T* + 1 post‐session wellness. (A) Predicted wellness score (colour) based on varying load range (Y‐axis) across the days of the season (X‐axis); (B) Derivative slope indicating rate of change in wellness across the external load range evaluated at the mean value of the temporal covariate. Grey shading represents 95% confidence intervals of the slope. Rug marks on X‐axis indicate the distribution of observed external load values.

Model performance for the next day wellness analyses showed deviance explained ranging from 63.1% to 66.2% (Table 2), indicating that a substantial proportion of the variance in next day wellness was explained by the training load metrics. Notably, although TD produced the highest *R* ^2^ value, the differences across metrics were minimal (Δ*R* ^*2*^ < 0.02). All GAM smooth terms were statistically significant (*p* < 0.001), and EDF values between 2.6 and 7.8 supporting non‐linear relationships for all metrics. HMLD's smooth had an EDF of 3.9, and HSRD 2.6, whereas TD had a significantly higher value of 7.8, indicating a more complex relationship between TD and wellness. The steeper colour gradient for HMLD in Figure 1A suggests a slightly stronger effect relative to the other metrics; however, all load metrics demonstrated a clear next‐day impact on player wellness.

#### 3.1.2 *T* + 2 Wellness Score

By 2 days post session, the relationship between wellness and all load metrics was much weaker than on *T* + 1; however, it remained negative (Figure 2). HMLD continued to show a decline with higher loads, but the effect was small (*β* = −0.0003, 95% CI [−0.0004, −0.0003]). HSRD showed a similar, effect (*β* = −0.0004, 95% CI [−0.0005, −0.0004]), which was marginally steeper than HMLD. These slope values suggest that even a large increase in either of these metrics, 2 days prior would only correspond to a very slight decrease in wellness, with athletes being nearer to baseline by *T* + 2. TD had no association with *T* + 2 (*β* = 0.0000, 95% CI [−0.0000, −0.0000]), indicating that there were no meaningful changes in wellness across the range of TD on *T* + 2. The same 300m increase in HMLD from *T* + 1 corresponded to changes of < 0.1 units in this time period. Figure 2B shows noticeably flatter curves, illustrating the above results, that wellness had become less responsive to training load after 48 hours.

**FIGURE 2:**
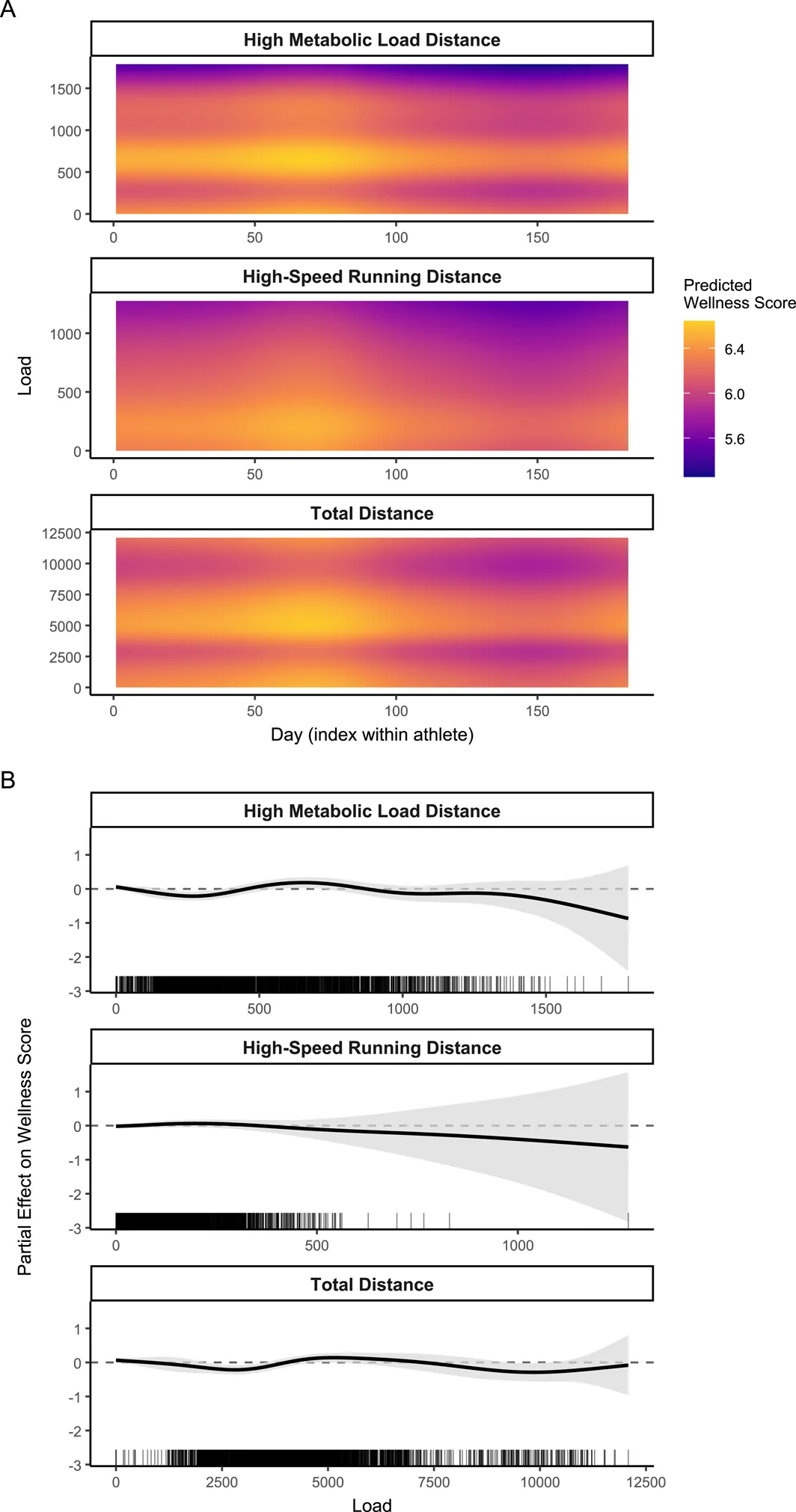
Generalised additive model outputs for *T* + 2 wellness. (A) Predicted wellness score (colour) based on varying load range (Y‐axis) across the days of the season (X‐axis); (B) Derivative slope indicating rate of change in wellness across the external load range evaluated at the mean value of the temporal covariate. Grey shading represents 95% confidence intervals of the slope. Rug marks on X‐axis indicate the distribution of observed external load values.

All models at this time point showed similar performance, with deviance explained between 62.1% and 62.3% (Table 2). All models had similar explanatory power at this time point. Despite relatively small effects, HMLD and TD both remained statistically significant (*p* < 0.001), indicating that the patterns were unlikely due to chance. This is in contrast to HSRD, which was not statistically significant (*p* = 0.110). EDF values for HMLD increased from *T* + 1, indicating more irregularity in the dose‐response relationship, as shown by the colour spectrum in Figure 2A, and the slope gradient in Figure 2B in comparison to the same visualisations in Figure 1. TD and HSRD had lower EDF values than *T* + 1, consistent with a more linear effect, as shown in the slope calculations.

#### 3.1.3 *T* + 3 Wellness Score

By 3 days post session, the relationship between load and wellness were minimal, consistent with near‐complete recovery from the session (Figure 3). HMLD had minimal impact on *T* + 3 wellness (*β* = 0.0001, 95% CI [0.0000, 0.0001]), displaying a similar relationship to TD (*β* = 0.0000, 95% CI [0.0000, 0.0000]), with distances not affecting changes in wellness score at this time point. HSRD showed an inverse positive relationship (*β* = 0.0006, 95% CI [0.0000, 0.0007]), however, this may have been influenced by sparse data at the high end of the load's range. A 300 m increase of HMLD at this time point was no longer associated with any meaningful change in wellness score. Figure 3B shows that the slopes for each of the metrics were mostly flat, with HMLD and HSRD showing a slight uptick in the highest loads.

**FIGURE 3:**
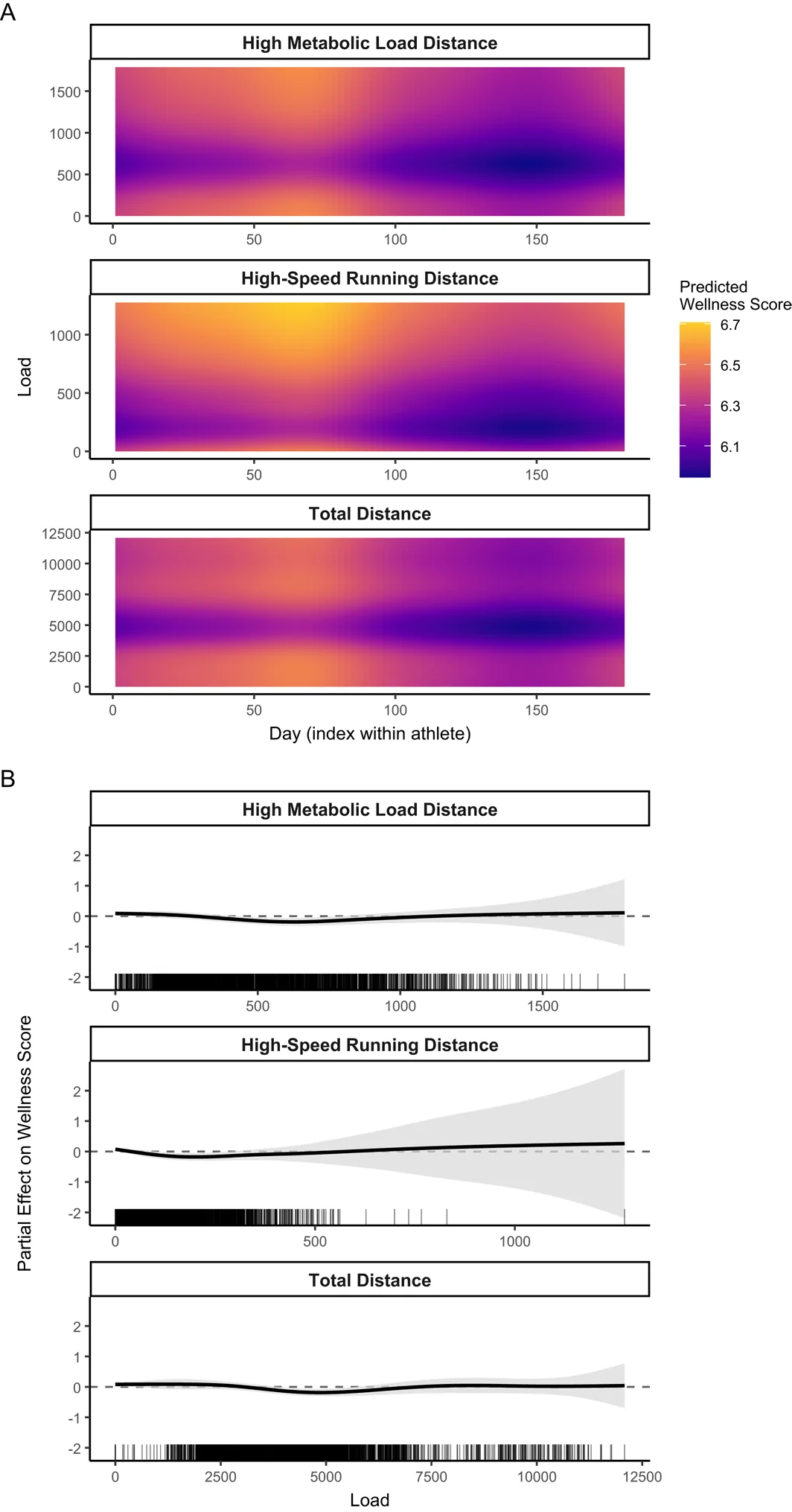
Generalised additive model outputs for *T* + 3 wellness. (A) Predicted wellness score (colour) based on varying load range (Y‐axis) across the days of the season (X‐axis); (B) Derivative slope indicating rate of change in wellness across the external load range evaluated at the mean value of the temporal covariate. Grey shading represents 95% confidence intervals of the slope. Rug marks on X‐axis indicate the distribution of observed external load values.

Models at *T* + 3 showed very similar behaviour between each of the metrics, with a deviance explained of 62.1% for all metrics. Despite minimal slopes for all metrics, the smooth terms remained statistically significant (*p* < 0.001). EDF for HMLD and TD decreased from *T* + 2 to *T* + 3, reflecting the near 0 relationship above. In contrast, HSRD had an increase in the EDF value, possibly due to the uptick in wellness at the highest loads (Figure 3). However, this should be interpreted with caution as relatively fewer observations were present at the upper end of this metric, as indicated by the rug plots (Figure 3B).

#### 3.1.4 Session Ratings of Perceived Exertion (sRPE)

When investigating the relationship with sRPE, all metrics displayed positive associations, indicating higher perceived exertion was linked to increase in external load (Figure 4). HMLD was associated with a strong increase (*β* = 0.0039, 95% CI [0.0038, 0.0040]), however, HSRD showed the largest slope, indicating the most pronounced effect on RPE (*β* = 0.0063, 95% CI [0.0063, 0.0064]), and TD the weakest (*β* = 0.0005, 95% CI [0.0005, 0.0005]). This slope indicated that with a 300m increase in HMLD, there is an approximate increase of 1–1.5 units of sRPE. Figure 4B for all metrics was relatively stable across their distance range, with HSRD being almost completely linear, indicating a consistent dose‐response pattern.

**FIGURE 4:**
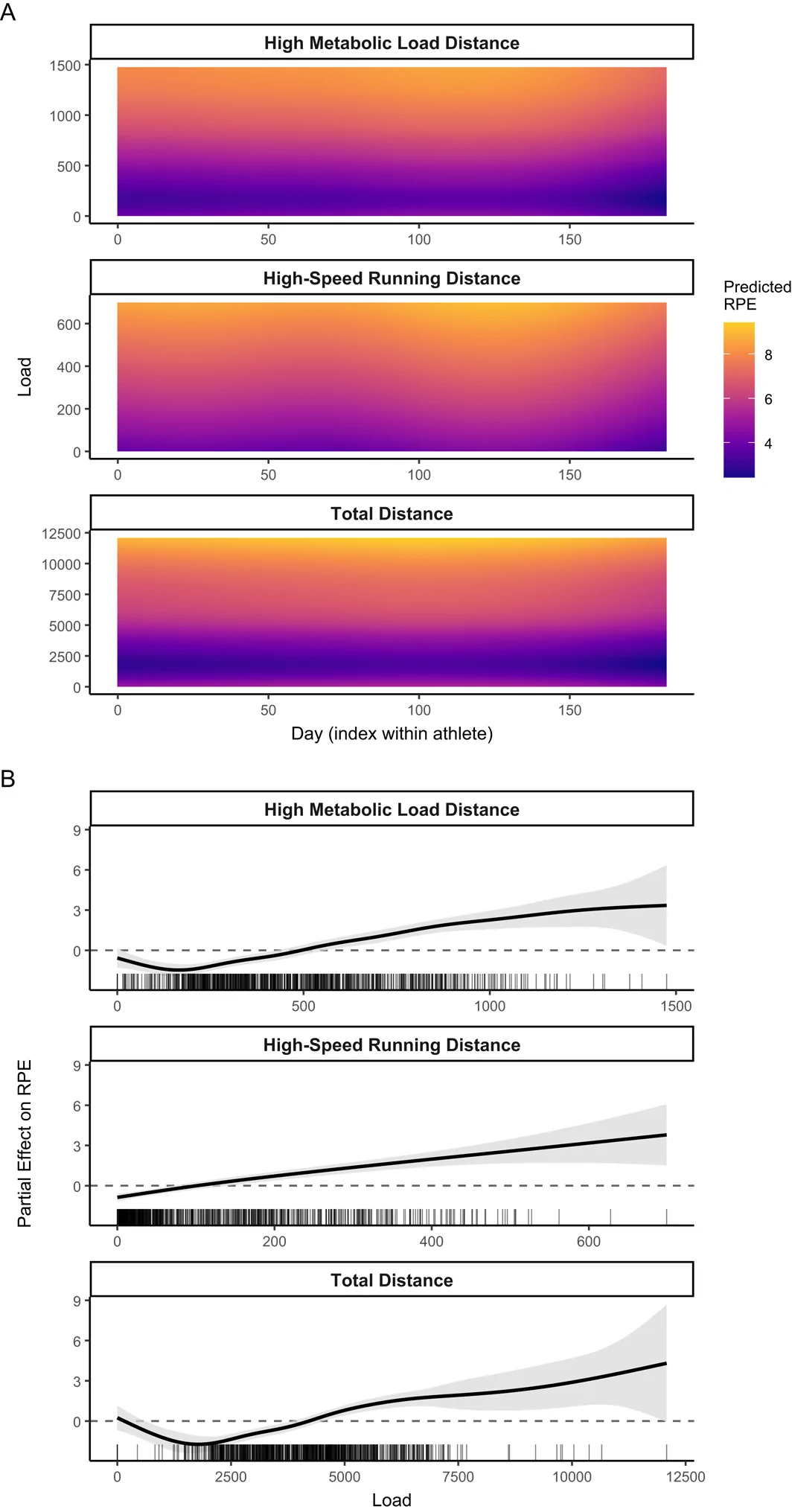
Generalised additive model outputs for sRPE. (A) Predicted wellness score (colour) based on varying load range (Y‐axis) across the days of the season (X‐axis); (B) Derivative slope indicating rate of change in sRPE across the external load range evaluated at the mean value of the temporal covariate. Grey shading represents 95% confidence intervals of the slope. Rug marks on X‐axis indicate the distribution of observed external load values.

Model performance was lower than for the wellness models, with deviance explained ranging from 39.0% to 51.2%. HMLD produced the strongest model fits (*R* ^2^ = 0.497), aligning with the clearer visual gradient changes in Figure 4A, while total distance and high‐speed running distance showed weaker explanatory power. All smooth terms were statistically significant (*p* < 0.001). EDF values were between 2.64–7.94, indicating non‐linearity overall, with HMLD and TD showing higher values, and a more pronounced curve in Figure 4B, compared to HSRD, which had a much more linear relationship.

#### 3.1.5 Wellness to Same‐Day Training

Finally, an important comparison to investigate is how wellness can inform athletes' same‐day external load‐derived training and game performance. All metrics were modelled for this relationship with results presented in Supporting Information S3: Table S1 and Supporting Information S2: Figures S6–S7; however, for brevity, only the HMLD results are discussed (Table 3; Figure 5). Motivation to train demonstrated a strong positive association with same‐day HMLD (*β* = 67.56, 95% CI [65.54, 70.58]). Positive relationships were also observed for fatigue (*β* = 50.50, 95% CI [47.44, 53.55]) and muscle soreness (*β* = 49.23, 95% CI [44.59, 53.87]), while mood (*β* = 32.89, 95% CI [32.89, 32.89]) and sleep quality (*β* = 32.98, 95% CI [31.51, 34.45]) showed smaller but still positive associations. In contrast, stress was negatively associated with same‐day HMLD (*β* = −10.58, 95% CI [−12.99, −8.17]). This relationship shows that a 0.3 unit increase in motivation corresponds with an increase in HMLD of ∼200m, whereas the same increase in stress equates to 30m less distance. Similar directional relationships were observed for TD and HSRD (Supporting Information S3: Table S1).

**FIGURE 5:**
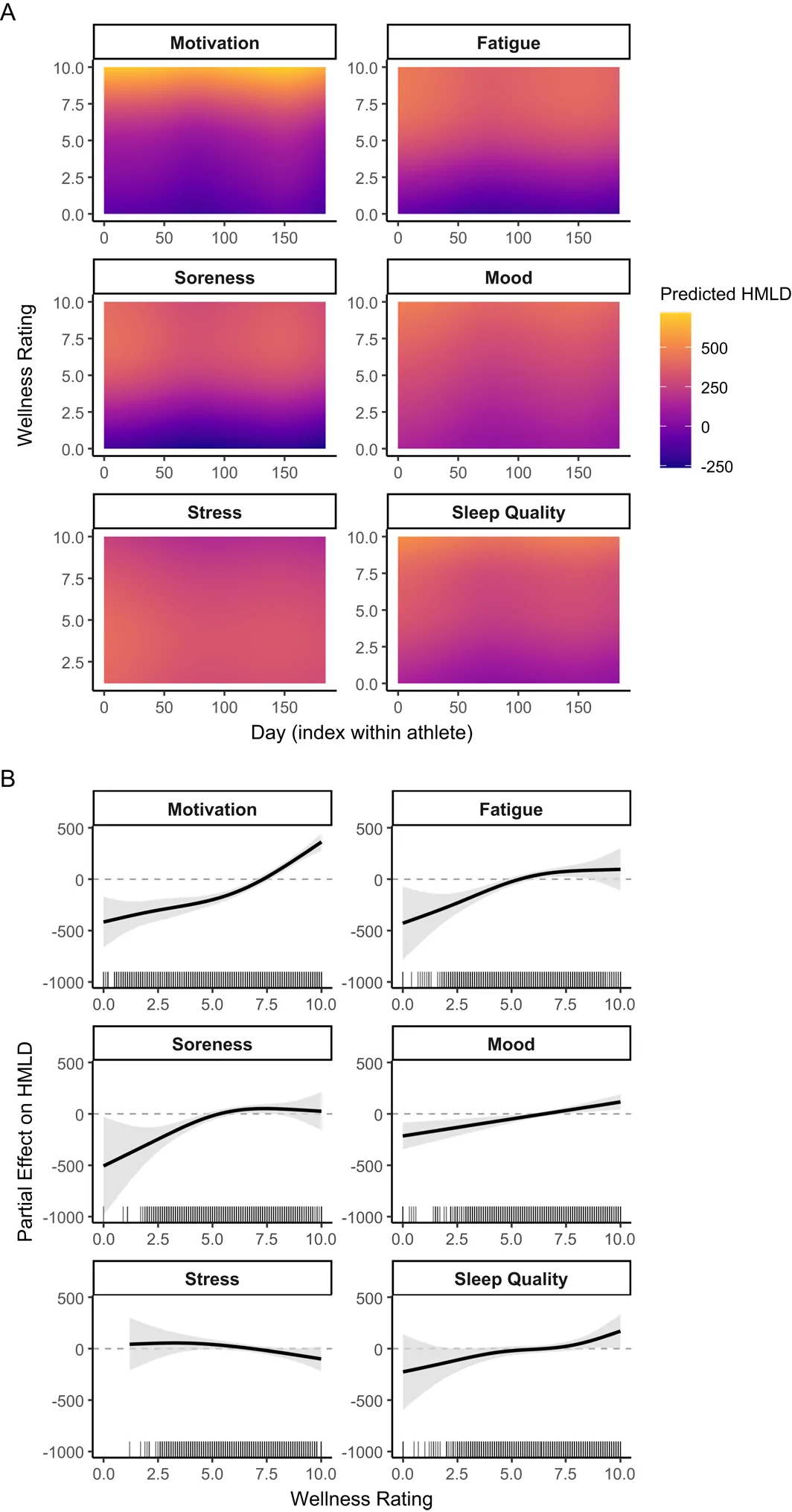
Generalised additive model outputs for wellness predicting HMLD. (A) Predicted HMLD (colour) based on varying load range (Y‐axis) across the days of the season (X‐axis); (B) Derivative slope indicating rate of change in HMLD across the wellness metric range evaluated at the mean value of the temporal covariate. Grey shading represents 95% confidence intervals of the slope. Rug marks on X‐axis indicate distribution of observed wellness values.

Model performance for this relationship showed adjusted *R* ^2^ values of 0.086 (Stress) to 0.215 (Motivation), with deviance explained between 9.2% and 22.1%. All smooth terms were statistically significant (*p* < 0.001), with EDF values between 1.0 (Mood) and 3.21 (Sleep Quality). Figure 5B confirms the linear relationship with mood, and non‐linearity for the other metrics as shown by EDF values. Similar relationships were found for TD and HSRD, with all associations smaller than HMLD, except the relationship between Motivation to Train and TD (Supporting Information S3: Table S2).

## 4 Discussion

This study examined the non‐linear relationship between three external load metrics‐total distance, high‐speed running distance, high metabolic load distance – and both athlete wellness (up to 3 days post‐session) and sRPE in an elite A‐League Women's football team. Higher training loads for each metric were generally associated with poorer next day wellness, with diminishing, but similar relationships by two and 3 days post‐session. HMLD generally demonstrated comparatively stronger and more stable associations with athlete‐reported wellness across each timepoint, whereas TD and HSRD showed weaker and less consistent associations (Figure 1, 2, 3). The relatively high explanatory performance observed in the wellness models should be interpreted within the context of the monitoring framework used, which incorporated participant‐level random effect, temporal structure and an autoregressive wellness adjustment, rather than reflecting the explanatory capacity of external load alone (Twisk 2013). In contrast, RPE demonstrated strong positive non‐linear associations with all three external load metrics, with the strongest relationships observed for HSRD and HMLD. The RPE models also showed higher curve flexibility than most wellness models, reflecting a more complex dose‐response behaviour within sessions. These findings suggest that HMLD demonstrated comparatively stronger associations with both the immediate perceptual load (sRPE) and short‐term changes in athlete‐reported wellness than TD or HSRD alone, within the context of training‐related demands.

Across the wellness models, the clearest relationships emerged the day after a training session, where all metrics demonstrated poorer next‐day wellness with increased load, consistent with expected short‐term neuromuscular and perceptual fatigue responses, and impacted sleep quality reported in team sports (Fullagar et al. 2015; Twist and Highton 2013). HMLD was associated with the greatest declines of wellness at this timepoint, along with the largest partial effects, aligning with the physiological rationale that frequent accelerations, decelerations and high‐intensity transitions carry substantial eccentric and metabolic cost (Harper et al. 2019). A 300 m increase in HMLD was associated with an approximate 0.3–0.4 unit reduction in next‐day wellness, which represents an observable change in athlete status. By *T*+2, the magnitude of these effects was significantly reduced, with comparable increases in HMLD corresponding to changes of < 0.1 wellness units. By *T*+3, slope estimates were near zero, indicating that variations of HMLD were no longer meaningfully associated with changes in wellness. These timelines align with previous evidence where reductions in strength, jump performance and neuromuscular function typically recover within 48–72 hours post‐match (Brownstein et al. 2017; Nedelec et al. 2014).

Session RPE increased as external load for all metrics increased. HMLD demonstrated the largest association, with an increase of 300m corresponding to an increased RPE of 1–1.5 units, indicating a perceptible rise of perceived effort. This pattern is consistent with the notion that high‐intensity running and rapid changes in direction can affect immediate perceptual strain (Tan et al. 2025a). The non‐linear nature of the sRPE response highlights that perceived exertion does not scale uniformly with external load. At lower loads, a larger increase in external load corresponded to greater increases in sRPE, whereas at higher loads, increases in external load resulted in smaller changes in sRPE. This pattern reflects a diminishing perceptual response at higher workload levels. Such behaviour is consistent with contemporary models of dose‐response relationships, where internal load does not scale linearly with increases in external demand (Impellizzeri et al. 2019).

Morning wellness demonstrated statistically significant, but limited associations with same‐day external load, suggesting that athlete‐reported wellness was moderately associated with the same‐day external load. Small changes in wellness corresponded to a measurable distance in load, with a 0.3 unit increase in motivation corresponding to a 200m increase in HMLD, while a comparable increase in fatigue resulted in a 150m decrease in HMLD. These findings suggest that athlete reported wellness was associated with same‐day external load, and HMLD was the most sensitive to these daily changes, demonstrating comparatively stronger relationships than HSRD or TD. This aligns with previous findings in elite football, where poorer morning wellness scores were associated with small but statistically significant reductions in same‐day HSRD and peak velocity (Malone et al. 2018).

When comparing the relative model performance of the three metrics, HMLD consistently explained the greatest proportion of variance across wellness and RPE models, and showed the most stable non‐linear patterns, when compared to TD and HSRD. This may reflect HMLD's integration of speed‐ and acceleration‐related energetic demands, although overlap exists between the selected external load metrics. In contrast, HSRD was less strongly associated with the self‐reported outcomes, potentially reflecting greater within‐player variability with individual speed band calculations, and contextual dependence of high‐speed actions. TD demonstrated stable but less specific associations across models, consistent with its broad but non‐specific representation of session load (Vanrenterghem et al. 2017).

Despite these results, this study should be interpreted with contextual consideration. As the analysis was conducted within a single A‐League Women's team, it provides strong ecological validity, but limits generalisability to other teams and competition levels. Therefore, the tactics of the investigated team may have influenced the amount of HSRD recorded, given the contextual dependence of high speed running actions in football. A further limitation is that HMLD was derived using the proprietary Catapult Sports implementation of metabolic power, with a manufacture‐defined threshold of 25.5 W/kg applied by the platform. As outlined previously, metabolic power‐derived external load metrics have been predominantly developed and applied within male football populations, with limited validation of the associated thresholds in elite female athletes. Sex differences have been reported in sprint and intermittent endurance capacities in elite football, with female players demonstrating lower values than male players (Cardoso de Araújo et al. 2020). These differences suggest that the application of the same fixed metabolic power threshold may not necessarily represent an equivalent relative physiological intensity in male and female athletes. Although this reflects the real‐world applied use of the system, the suitability of fixed absolute metabolic power thresholds for elite women's football requires further research. Between‐athlete heterogeneity in fitness, position and match exposure may have also influenced observed associations. Additionally, field‐based running metrics do not capture all training and recovery stressors such as resistance training, or external psychosocial influences, which may have contributed to subsequent wellness responses, independent of quantified training load. The subjective nature of wellness and RPE, although commonly used in elite sport, is also susceptible to perceptual bias and contextual influences unrelated to training load. The use of a composite wellness score may show reduced changes in load‐sensitive measures such as fatigue or muscle soreness because of averaging with measures less influenced by training load. Finally, although GPS‐derived metrics provide valuable insight into running performance, minor differences in load should be interpreted cautiously given the inherent between‐unit measurement variability. Future research should explore whether the non‐linear patterns observed are consistent across multiple teams and competition levels, whether similar relationships appear when integrating objective markers such as heart rate variability, and whether sex‐specific or individualised metabolic power thresholds improve the interpretation of high‐intensity external load in women's football.

### 4.1 Practical Implications

For practitioners, HMLD reflects the mechanical and metabolic stresses associated with accelerations, decelerations and high‐intensity movements. In the present study, it demonstrated comparatively stronger and more consistent associations with self‐report measures than the other examined metrics, and may represent a useful load metric for routine athlete monitoring. From an applied perspective, this suggests that practitioners may benefit from incorporating intensity‐derived metrics alongside the traditional volume‐ and speed‐based summaries in session load interpretations. This study showed that a typical between‐session difference of 300m in HMLD was associated with observable changes in next‐day wellness (0.3–0.4 unit decrease) and sRPE (1–1.5 unit increase), providing a practical reference for interpreting whether a session may require increased recovery demands. Such thresholds may be particularly useful when reviewing congested training periods or evaluating the cumulative impact of high‐intensity training sessions across a schedule. Athlete wellness was also associated with same‐day GPS‐derived running output, with athletes reporting higher motivation or lower fatigue accumulating greater HMLD during training. This suggests that HMLD may also be sensitive to day‐to‐day differences in athlete‐reported readiness. If practitioners are restricted to monitoring only a small number of external load variables, HMLD may therefore provide a useful summary representation of intensity‐derived load while maintaining the sensitivity needed to detect meaningful changes in load, wellness and perceived exertion. Used in this manner, HMLD may support more informed decisions regarding session progression, recovery management and individual load adjustments across the training week.

## 5 Conclusion

In summary, this study demonstrated non‐linear relationships between external load metrics and both subsequent days' wellness and same‐day RPE in an elite women's football environment. HMLD generally demonstrated the strongest associations compared to traditional distance‐based measures (TD and HSRD), with both immediate perceptual exertion and short‐term athlete‐reported wellness. Load‐related effects on wellness were strongest the next day, with effects substantially diminishing two to 3 days post session towards baseline, suggesting that most wellness responses had largely attenuated from that training session's demands in that time period. Athlete wellness was also associated with the same‐day GPS‐derived training load, with day‐to‐day differences in wellness associated with measurable changes in HMLD. These findings highlight the value of intensity‐derived metrics such as HMLD within applied settings as practical and informative measures for monitoring the interaction between training load, athlete wellness, and perceived exertion in elite women's football.

## Author Contributions

**Joshua D. Waugh:** conceptualisation, methodology, investigation, data curation, formal analysis, writing – original draft, writing – review and editing. **Kate L. Pumpa:** supervision, writing – review and editing. **Celeste E. Coltman:** supervision, writing – review and editing. **Nick Ball:** supervision, writing – review and editing. **Bradley Clark:** supervision, writing – review and editing. **Jocelyn K. Mara:** conceptualisation, methodology, supervision, writing – review and editing.

## Funding

This research was supported by the 50:50 Women in Sports Scholarship from the University of Canberra and Capital Football via the Australian Government Research Training Program. No additional funding was provided for the study's execution.

## Ethics Statement

Ethical approval was obtained from the University of Canberra Human Research Ethics Committee (HREC ID: 14,242).

## Consent

All participants provided informed consent to participate in the research. All participants provided informed consent for the publication of anonymised data.

## Conflicts of Interest

The primary author of this study held an embedded performance role with the participating team as part of their doctoral research. To minimise any potential bias, participant recruitment was conducted by co‐authors independent of the team. The authors affirm that this professional association did not influence the study design, analysis, interpretation of data, or reporting of findings. No other conflicts of interest are declared.

## Supporting information


Supporting Information S1

Supporting Information S2

Supporting Information S3

## Data Availability

Due to the nature of this research and the confidentiality of participants' identities, the data are not publicly available.
