# Supplementary material for: Running the Numbers: High Metabolic Load Distance as an Alternative to Traditional External Load Metrics in Elite Women's Football
Source: Eur J Sport Sci. 2026 Nov 13;26(12):e70288. doi: 10.1002/ejsc.70288 (PMC13766684; doi:10.1002/ejsc.70288)
Supplement: Supplementary file 1 [file EJSC-26-e70288-s001.docx]

**Supplementary Figure 1.** Pearson’s correlation matrix of external load metrics analysed

**Supplementary Figure 2**. Generalised additive model outputs for T + 1 post-session wellness for outfield players

*Note: A) Predicted wellness score (colour) based on varying load range (Y-axis) across the days of the season (X-axis); B) Derivative slope indicating rate of change in wellness across the external load range evaluated at the mean value of the temporal covariate. Grey shading represents 95% confidence intervals of the slope. Rug marks on x-axis indicate distribution of observed external load values.*

**Supplementary Figure 3**. Generalised additive model outputs for T + 2 post-session wellness for outfield players

*Note: A) Predicted wellness score (colour) based on varying load range (Y-axis) across the days of the season (X-axis); B) Derivative slope indicating rate of change in wellness across the external load range evaluated at the mean value of the temporal covariate. Grey shading represents 95% confidence intervals of the slope. Rug marks on x-axis indicate distribution of observed external load values.*

**Supplementary Figure 4**. Generalised additive model outputs for T + 3 post-session wellness for outfield players

*Note: A) Predicted wellness score (colour) based on varying load range (Y-axis) across the days of the season (X-axis); B) Derivative slope indicating rate of change in wellness across the external load range evaluated at the mean value of the temporal covariate. Grey shading represents 95% confidence intervals of the slope. Rug marks on x-axis indicate distribution of observed external load values.*

**Supplementary Figure 5.** Generalised additive model outputs for sRPE for outfield players.

*Note: A) Predicted wellness score (colour) based on varying load range (Y-axis) across the days of the season (X-axis); B) Derivative slope indicating rate of change in sRPE across the external load range evaluated at the mean value of the temporal covariate. Grey shading represents 95% confidence intervals of the slope. Rug marks on x-axis indicate distribution of observed external load values.*

**Supplementary Figure 6**. Predicted external load (colour) based on varying wellness range (Y-axis) across the days of the season (X-axis)

*Note: A) High Metabolic Load Distance; B) High Speed Running Distance; C) Total Distance*

**Supplementary Figure 7**. Derivative slope indicating rate of change in wellness across the external load range evaluated at the mean value of the temporal covariate. Grey shading represents 95% confidence intervals of the slope. Rug marks on x-axis indicate distribution of observed external load values.

*Note: A) High Metabolic Load Distance; B) High Speed Running Distance; C) Total Distance*
