# Supplementary material for: Running the Numbers: High Metabolic Load Distance as an Alternative to Traditional External Load Metrics in Elite Women's Football
Source: Eur J Sport Sci. 2026 Nov 13;26(12):e70288. doi: 10.1002/ejsc.70288 (PMC13766684; doi:10.1002/ejsc.70288)
Supplement: Supplementary file 2 [file EJSC-26-e70288-s002.docx]

\# Supplementary File 1. Annotated illustrative R Code for

\# generalised additive monitoring (GAM) analysis

\#

\# ============================================================

\# SUPPLEMENTARY MATERIAL: ANNOTATED R ANALYSIS CODE

\# ============================================================

\#

\# This code provides an illustrative example of the

\# generalised additive modelling approach used to examine

\# associations between external load and subsequent wellness.

\#

\# The example uses High Metabolic Load Distance (HMLD) as the

\# predictor and wellness score as the response variable.

\#

\# Athlete-level data and data-management procedures are not

\# provided. The code assumes that the dataset has already

\# been prepared for statistical analysis.

\#

\# The example is not intended to reproduce the numerical

\# results of the study without the underlying dataset,

\# which cannot be supplied due to privacy of participants.

\#

\# Please note that the code will need to be copied into a RStudio and R to work.

\#

\# ============================================================

\# 1. REQUIRED PACKAGE

\# ============================================================

library(mgcv)

\# ============================================================

\# 2. MODEL VARIABLES

\# ============================================================

\#

\# The prepared dataset 'd' contains:

\#

\# .outcome

\#	Wellness score at the selected outcome horizon

\#	(one day following the external load observation).

\#

\# high_metabolic_load_distance

\#	Daily HMLD recorded by the GPS system.

\#

\# day_index

\#	Temporal index representing days within each athlete.

\#

\# player_name

\#	Participant identifier used as a random effect.

\#

\# wellness_score_lag1

\#	Wellness score from the preceding observation.

\#

\# wellness_score_lag2

\#	Wellness score from two preceding observations.

\#

\# wellness_score_ar_decay3

\#	Weighted combination of three preceding wellness scores:

\#	lag1 + (1/3 * lag2) + (1/9 * lag3).

\#

\# These variables were generated during data preparation and

\# data wrangling procedures

\# ============================================================

\# 3. SMOOTH BASIS DIMENSIONS

\# ============================================================

\#

\# Basis dimensions were selected according to the number

\# of unique observations, with a maximum of 10.

k_load_raw <- length(

unique(d$high_metabolic_load_distance)

)

k_load <- max(3, min(10, k_load_raw - 1))

k_day_raw <- length(unique(d$day_index))

k_day <- max(3, min(10, k_day_raw - 1))

\# ============================================================

\# 4. GENERALISED ADDITIVE MODEL

\# ============================================================

\#

\# The model includes:

\#

\# - A smooth term for HMLD.

\# - A smooth term for temporal variation.

\# - A participant-specific random intercept.

\# - Lagged wellness terms.

\#

\# Model parameters were estimated using restricted maximum

\# likelihood (REML).

\#

\# Missing observations were omitted during model fitting.

model_formula <- as.formula(

paste0(

".outcome ~ ",

"s(high_metabolic_load_distance, k = ", k_load, ") + ",

"s(day_index, k = ", k_day, ") + ",

"s(player_name, bs = 're') + ",

"wellness_score_lag1 + ",

"wellness_score_lag2 + ",

"wellness_score_ar_decay3"

)

)

model <- mgcv::gam(

formula = model_formula,

data = d,

method = "REML",

na.action = na.omit

)

\# ============================================================

\# 5. MODEL SUMMARY

\# ============================================================

\#

\# Extract model fit statistics, smooth-term estimates and

\# parametric coefficients.

model_summary <- summary(model)

print(model_summary)

\# Parametric coefficients, including lagged wellness terms

model_summary$p.table

\# Smooth-term statistics, including effective degrees of

\# freedom, F-statistics and p-values

model_summary$s.table

\# Explained deviance and adjusted R-squared

model_summary$dev.expl

model_summary$r.sq

\# ============================================================

\# 6. MODEL DIAGNOSTICS

\# ============================================================

\#

\# Examine residual behaviour and assess whether the

\# specified smooth basis dimensions are adequate.

mgcv::gam.check(model)

\# ============================================================

\# 7. PARTIAL EFFECT OF HMLD

\# ============================================================

\#

\# Extract the estimated partial effect of HMLD while

\# accounting for the other terms included in the model.

\#

\# Approximate uncertainty bounds are calculated using

\# two standard errors above and below the fitted effect.

hml_effect <- mgcv::plot.gam(

model,

select = 1,

seWithMean = TRUE,

plot = FALSE

)[[1]]

effect_df <- data.frame(

hml_distance = hml_effect$x,

fit = hml_effect$fit,

lower = hml_effect$fit - 2 * hml_effect$se,

upper = hml_effect$fit + 2 * hml_effect$se

)

\# ============================================================

\# 8. VISUALISATION

\# ============================================================

\#

\# Plot the adjusted HMLD association and approximate

\# uncertainty interval.

library(ggplot2)

ggplot(

effect_df,

aes(x = hml_distance, y = fit)

) +

geom_hline(

yintercept = 0,

linetype = "dashed"

) +

geom_ribbon(

aes(ymin = lower, ymax = upper),

alpha = 0.2

) +

geom_line() +

labs(

x = "High Metabolic Load Distance",

y = "Partial Effect on Wellness Score"

) +

theme_classic()

\# ============================================================

\# END OF ANNOTATED CODE EXAMPLE

\# ============================================================

\#

\# The complete analysis applied the modelling framework

\# across multiple external load metrics and outcomes.

\#

\# Wellness outcomes were examined across one-, two- and

\# three-day horizons, as well as predicting same-day load.

\# Session RPE was examined separately using same-day models

\# without lagged outcome terms.
