# Supplementary material for: Running the Numbers: High Metabolic Load Distance as an Alternative to Traditional External Load Metrics in Elite Women's Football
Source: Eur J Sport Sci. 2026 Nov 13;26(12):e70288. doi: 10.1002/ejsc.70288 (PMC13766684; doi:10.1002/ejsc.70288)
Supplement: Supplementary file 3 [file EJSC-26-e70288-s003.docx]

**Supplementary Table 1.** Calculations of Average Slope Derivatives

| **Model** | **Average Slope Estimate** | **Slope SE** | **Sloper Lower CI** | **Slope Upper CI** |
| --- | --- | --- | --- | --- |
| *T + 1 Wellness* |  |  |  |  |
| Total Distance - Effect Size | -0.0001 | 0.0000 | -0.0001 | -0.0001 |
| High-Speed Running Distance - Effect Size | -0.0006 | 0.0000 | -0.0007 | -0.0006 |
| High Metabolic Load Distance - Effect Size | -0.0011 | 0.0000 | -0.0012 | -0.0011 |
| Total Distance - Seasonal Trend | -0.0024 | 0.0001 | -0.0026 | -0.0021 |
| High-Speed Distance - Seasonal Trend | -0.0023 | 0.0001 | -0.0026 | -0.0020 |
| High Metabolic Load Distance - Seasonal Trend | -0.0023 | 0.0001 | -0.0025 | -0.0020 |
| *T + 2 Wellness* |  |  |  |  |
| Total Distance - Effect Size | 0.0000 | 0.0000 | 0.0000 | 0.0000 |
| High-Speed Running Distance - Effect Size | -0.0004 | 0.0000 | -0.0005 | -0.0004 |
| High Metabolic Load Distance - Effect Size | -0.0003 | 0.0000 | -0.0004 | -0.0003 |
| Total Distance - Seasonal Trend | -0.0022 | 0.0002 | -0.0025 | -0.0018 |
| High-Speed Distance - Seasonal Trend | -0.0023 | 0.0002 | -0.0026 | -0.0019 |
| High Metabolic Load Distance - Seasonal Trend | -0.0021 | 0.0002 | -0.0025 | -0.0018 |
| *T + 3 Wellness* |  |  |  |  |
| Total Distance - Effect Size | 0.0000 | 0.0000 | 0.0000 | 0.0000 |
| High-Speed Running Distance - Effect Size | 0.0006 | 0.0000 | 0.0000 | 0.0007 |
| High Metabolic Load Distance - Effect Size | 0.0001 | 0.0000 | 0.0000 | 0.0001 |
| Total Distance - Seasonal Trend | -0.0020 | 0.0002 | 0.0000 | -0.0017 |
| High-Speed Distance - Seasonal Trend | -0.0020 | 0.0002 | 0.0000 | -0.0017 |
| High Metabolic Load Distance - Seasonal Trend | -0.0020 | 0.0002 | 0.0000 | -0.0017 |
| *Same Day RPE* |  |  |  |  |
| Total Distance | 0.0005 | 0.0000 | 0.0005 | 0.0005 |
| High-Speed Running Distance | 0.0063 | 0.0000 | 0.0063 | 0.0064 |
| High Metabolic Load Distance | 0.0039 | 0.0001 | 0.0037 | 0.0040 |
| *Wellness to Same-Day HMLD Load* |  |  |  |  |
| Motivation to Train | 67.56 | 1.52 | 64.54 | 70.58 |
| Muscle Soreness | 49.23 | 2.34 | 44.59 | 53.87 |
| Mood | 32.89 | 0.00 | 32.89 | 32.89 |
| Stress | -10.58 | 1.21 | -12.99 | -8.17 |
| Fatigue | 50.50 | 1.54 | 47.44 | 53.55 |
| Sleep Quality | 32.98 | 0.74 | 31.51 | 34.45 |
| *Wellness to Same-Day Total Distance* |  |  |  |  |
| Motivation to Train | 631.47 | 12.67 | 606.33 | 656.61 |
| Muscle Soreness | 410.09 | 17.85 | 374.67 | 445.50 |
| Mood | 270.54 | 0.00 | 270.53 | 270.54 |
| Stress | -55.09 | 13.68 | -82.24 | -27.94 |
| Fatigue | 435.18 | 11.88 | 411.60 | 458.77 |
| Sleep Quality | 191.76 | 0.01 | 191.73 | 191.78 |
| *Wellness to Same-Day High-Speed Running Distance* |  |  |  |  |
| Motivation to Train | 16.81 | 0.35 | 16.11 | 17.51 |
| Muscle Soreness | 10.68 | 0.65 | 9.40 | 11.96 |
| Mood | 7.20 | 0.00 | 7.20 | 7.20 |
| Stress | -5.51 | 0.07 | -5.66 | -5.37 |
| Fatigue | 12.31 | 0.57 | 11.18 | 13.44 |
| Sleep Quality | 4.34 | 0.00 | 4.34 | 4.34 |

**Supplementary Table 2.** Descriptive Statistics of GAM analysis for the association between wellness and same-day external load.

| **Model** | **Adjusted R-Squared** | **Deviance Explained (%)** | **Sample Size (n)** | **edf** | **F-statistic** | **p-value** |
| --- | --- | --- | --- | --- | --- | --- |
| *High Metabolic Load Distance* |  |  |  |  |  |  |
| Motivation to Train | 0.215 | 0.221 | 3742 | 3.20 | 171.00 | 0.000 |
| Muscle Soreness | 0.100 | 0.106 | 3742 | 3.03 | 23.48 | 0.000 |
| Mood | 0.094 | 0.099 | 3742 | 1.00 | 59.53 | 0.000 |
| Stress | 0.086 | 0.092 | 3742 | 2.62 | 7.06 | 0.000 |
| Fatigue | 0.110 | 0.116 | 3742 | 2.83 | 35.64 | 0.000 |
| Sleep Quality | 0.091 | 0.097 | 3742 | 3.21 | 11.48 | 0.000 |
| *Total Distance* |  |  |  |  |  |  |
| Motivation to Train | 0.227 | 0.232 | 3742 | 3.30 | 217.02 | 0.000 |
| Muscle Soreness | 0.077 | 0.083 | 3742 | 3.00 | 28.27 | 0.000 |
| Mood | 0.068 | 0.073 | 3742 | 1.00 | 61.23 | 0.000 |
| Stress | 0.060 | 0.066 | 3742 | 3.05 | 7.47 | 0.000 |
| Fatigue | 0.089 | 0.095 | 3742 | 2.83 | 43.33 | 0.000 |
| Sleep Quality | 0.061 | 0.066 | 3742 | 1.01 | 29.51 | 0.000 |
| *High-Speed Running Distance* |  |  |  |  |  |  |
| Motivation to Train | 0.119 | 0.125 | 3742 | 2.68 | 91.83 | 0.000 |
| Muscle Soreness | 0.058 | 0.064 | 3742 | 2.87 | 11.63 | 0.000 |
| Mood | 0.053 | 0.059 | 3742 | 1.00 | 23.63 | 0.000 |
| Stress | 0.051 | 0.057 | 3742 | 1.50 | 7.05 | 0.001 |
| Fatigue | 0.066 | 0.072 | 3742 | 2.98 | 19.60 | 0.000 |
| Sleep Quality | 0.050 | 0.056 | 3742 | 1.01 | 8.23 | 0.004 |
